# Intact animacy perception during chase detection in ASD

**DOI:** 10.1038/s41598-017-12204-x

**Published:** 2017-09-19

**Authors:** Steven Vanmarcke, Sander van de Cruys, Pieter Moors, Johan Wagemans

**Affiliations:** 1 Brain and Cognition, KU Leuven, Leuven, 3000 Belgium; 2 Leuven Autism Research (LAuRes), KU Leuven, Leuven, 3000 Belgium

## Abstract

We explored the strength of implicit social inferences in adolescents with and without Autism Spectrum Disorder (ASD) using a chasing paradigm in which participants judged the absence/presence of a chase within a display of four seemingly randomly moving dots. While two of these dots always moved randomly, the two others could fulfill the role of being either the chasing (wolf) or chased (sheep) dot. In the chase-present (but not the chase-absent) trials the wolf displayed chasing behavior defined by the degree to which the dot reliably moved towards the sheep (chasing subtlety). Previous research indicated that chasing subtlety strongly influenced chase detection in typically developing (TD) adults. We intended to replicate and extend this finding to adolescents with and without ASD, while also adding either a social or a non-social cue to the displays. Our results confirmed the importance of chasing subtlety and indicated that adding social, but not non-social, information further improved chase detection performance. Interestingly, the performance of adolescents with ASD was less dependent on chasing subtlety than that of their TD counterparts. Nonetheless, adolescents with and without ASD did not differ in their use of the added social (or non-social) cue.

## Introduction

Autism Spectrum Disorder (ASD) represents a broad set of early onset neurodevelopmental psychiatric disorders that are characterized by two main symptom clusters. Firstly, individuals with ASD show persistent deficits in reciprocal social communication and social interaction. Secondly, individuals with ASD have restricted and repetitive patterns of behavior, interests or activities^1^. These symptoms are represented by atypicalities in developmental areas such as speech, language, sensory processing or relationship building. Furthermore, several studies have found individuals with ASD to be impaired on tasks assessing their knowledge of the mental states of other human beings (“mentalizing”) while sparing other perceptual and cognitive abilities (for review, see^2^). Overall, these deviations in the processing of social information are in line with the Theory of Mind (ToM) account of ASD^2,3^. ToM refers to the socio-cognitive ability to infer mental states of oneself and others implicitly, to interpret and understand their behaviors, and to guide one’s own actions accordingly. The theory describes a broad cluster of socio-communicative skills, which are assumed to be generally impaired in individuals with ASD. One of these skills relates to the processing and/or identification of (non-)verbal communicative behaviors^4,5^. Individuals with ASD are generally found to give less preferential attention to social objects and events (e.g., faces, humans, and social actions)^6,7^. In addition, other studies observed clear emotion recognition deficits in individuals with ASD (compared with typically developing (TD) participants) in tasks tapping into more complex socio-cognitive abilities such as the discrimination of more subtle emotional expressions^8,9^. Nonetheless, standardized, laboratory-controlled studies on social cognitive processing in ASD have yielded very inconsistent results due to various reasons^3^: (1) heterogeneity in the tested participant samples, (2) large stimulus and task variability making across-study generalization difficult, and (3) ambiguous experimental ToM designs allowing for non-social heuristics to solve the given task.

The discrepancy between intact or minimally impaired ToM task performance and impaired social adaptation observed in more naturalistic settings^10,11^ suggests that we need to develop alternative ToM operationalizations that can measure social deficits more sensitively. In the current study, we will therefore focus on a well-controlled, promising paradigm, using animated displays consisting of simple moving shapes, which appear to engage in animate and goal-directed behavior when presented to observers^12,13^. This automatic and effortless percept of shapes as interacting in social relationships is termed animacy perception and the interpretation of entities as intentional agents represents a constitutive component of the ability to spontaneously grasp social meanings^14^. In ASD, compared to TD participants, the subjective evaluation of animacy-relevant physical stimulus properties seems intact (e.g., observing trial-by-trial variations in motion complexity), while spontaneous neural responses to social cues, related to intrinsic salience and mentalistic inferences, are diminished^15^. Furthermore, previous research^16–18^ indicated that individuals with ASD used shorter, and less complex, verbal descriptions of social elements in animated displays of simple moving shapes similar to those pioneered by Heider and Simmel^12^. These results suggest that individuals with ASD appear to have problems in correctly describing the object- or goal-directedness of the observed activity. This was also evident in reduced monitoring of, and responsivity to, social cues signaling goal-directedness in children with ASD^19^, even when these cues were generated by simple, moving, geometric shapes depicting social interactions^20^. Nonetheless, these findings in participants with ASD did not result from poorer perceptual representations of other people’s actions due to dysfunctional visual circuitry, but from attentional atypicalities when processing socially relevant stimuli^21^. They were not restricted to social attention but also involved domain-general attention problems^22^, possibly related to a tendency to perseverate on images/situations of interest, exploring them in a more detail-oriented manner^23^.

Importantly, these differences in social attention between individuals with or without ASD often remain subtle and only become more prominent in more automatic, implicit measurements of attentional priority for social or non-social cues in naturalistic visual information^24^. Problematically, most studies using animated displays in ASD still focus on verbal self-report, requiring active self-reflection and conscious semantic labeling to complete the task, while not probing more implicit measures of agency detection^25^. Such a distinction between implicit and explicit forms of mentalizing has played a crucial role in clarifying the deficits in social cognition that occur in ASD. More specifically, we can hypothesize that individuals with ASD may deviate from typical social cognition in that mental states are not as readily experienced ‘directly’ (i.e., as given in our perceptual experience of others’ behavior), but rather that awareness of others’ mental states tends to require the (explicit) interpretation or reasoning that TD individuals usually reserve for social situations that are unusually ambiguous^26,27^. Such an interpretation is in line with ToM and, more recently, the Enactive Mind (EM) hypothesis^28^. This hypothesis argues that the process of acquiring embodied social cognition is derailed in the early development of children with ASD, due to a reduced salience of social stimuli and concomitant enactment of socially irrelevant aspects of the environment. Interestingly, previous studies found that children with ASD, compared to their TD counterparts, have a lower tendency to gaze at faces, e.g during free play^29,30^, and spend more time watching objects within the environment^31^. More recently, it was also reported that children with ASD directed their gaze further down and explored their lateral field of view more extensively than TD children^32^. In addition, a recent study noted that interactive and dynamic stimuli might be better than static displays to predict how children behave in real-life situations^33^. The authors thereby reasoned that human figures or faces, depicted in isolation, are only ‘social’ in the sense that they represent social beings, but not in terms of depicting (realistic) social behavior. *Reduced* attention to social interactions, in the presence of relatively intact ‘social attention’ to static or even dynamic individual faces, might suggest a greater deficit in attending to social actions rather than just social beings.

In order to further explore these implicit differences in social attention, we used a dynamic chase detection paradigm, similar to the paradigm of Gao and colleagues^13^ in TD participants, in which we specifically investigated the strength of implicit social inferences in adolescents with and without ASD. In this task, one geometrical shape (referred to as ‘wolf’) is chasing another (referred to as ‘sheep’), and this chasing behavior was defined by the degree to which the wolf reliably moved in the direction of the sheep, mimicking or deviating from perfectly ‘heat-seeking’ behavior by manipulating the maximal angular deviation of the heading of the wolf (chasing subtlety). Findings in TD participants indicated that this cue strongly determined chase detection performance and that, when the wolf’s motion only deviates slightly from perfect heat-seeking, chase detection already becomes much harder. Note that the perception of chasing of the sheep by the wolf is a spontaneous interpretation of animacy, agency and intentionality, which is based on the relative spatio-temporally defined trajectories between dots that are inherently inanimate. In the case of random, independent trajectories, all dots are perceived as randomly moving entities. In the case of a chase trial, the two trajectories that are yoked are automatically perceived as animate agents, one intentionally chasing the other. It is this automatic social interpretation of two interdependent trajectories which is interesting as a case of spontaneous social interpretations while not strictly being necessary to carry out the task.

**Figure 1:**
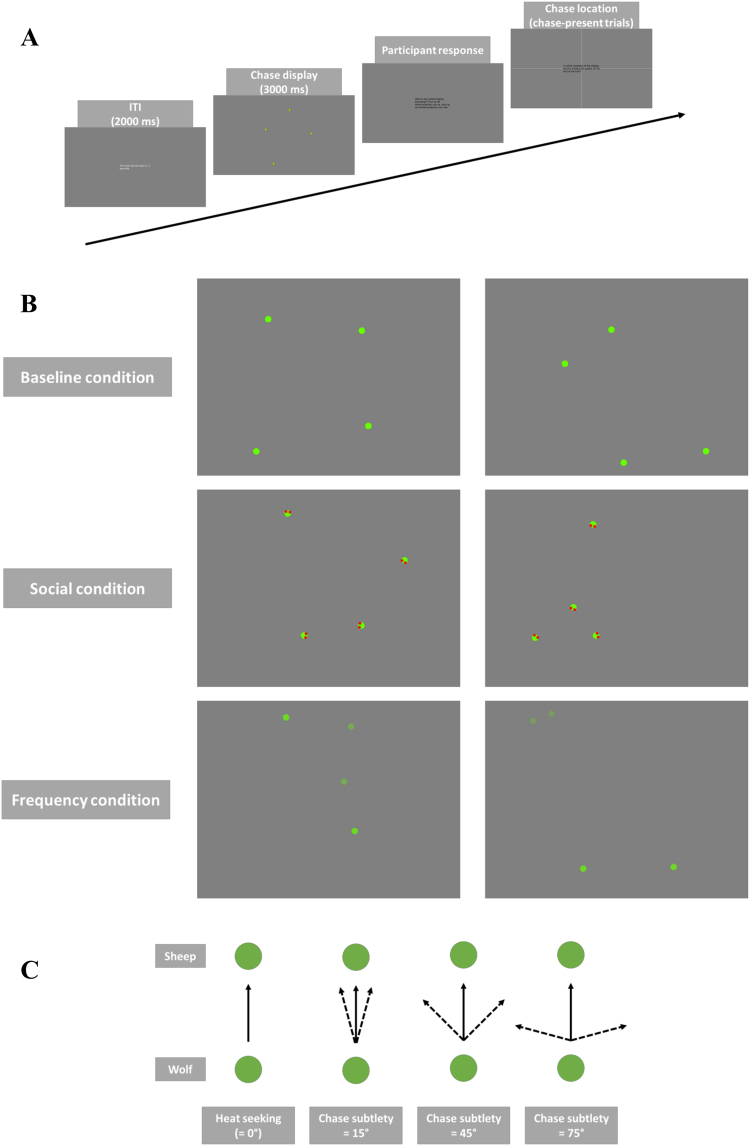
(**A**) Graphical overview of the trial design. (**B**) Static screen shots of the different task conditions. In the *baseline condition*, only the chasing subtlety was manipulated. In the *social condition*, eyes were added to all shapes and those of the wolf were always focused on the sheep in chase-present trials. In the *non-social condition*, the contrast of the shapes was manipulated periodically. A movie of the task is available on http://www.gestaltrevision.be/en/resources/supplementary-material. (**C**) Illustration of the chasing subtlety manipulation, implemented by manipulating the maximal angular deviation of the heading of the wolf compared to perfect “heat-seeking” behavior. When the chasing subtlety was 15° (or 45° or 75°), the wolf was always heading in the general direction of the sheep, but was not perfectly heat-seeking. The dot could move in any direction within a 30° (or 90° or 150^°^) window, with the window always centered on the (moving) sheep.

Furthermore, in a follow-up study^34^ the authors assessed, by interrupting the wolf’s chasing behavior periodically by different types of non-chasing motion, how (1) the detection of chasing is determined by the character and temporal grouping of ‘pursuit’ over time and (2) how these temporal dynamics can lead the visual system to either construct or actively reject interpretations of chasing. They concluded that the subtlety of the stimulus features that modulated agency detection in this task indicated that our awareness of animacy is not necessarily dependent on the conscious, deliberate appraisal of the stimuli, but is instead a purely perceptual process^35^. In our study, we intended to replicate the findings of Gao and colleagues^13^ on the influence of chasing subtlety on chase detection in TD participants (baseline condition). In this baseline condition, participants had to judge the absence or presence of a chase within a display of four seemingly randomly moving dots. While two of these dots always moved randomly, the two others could fulfill the role of being either the chasing (wolf) or chased (sheep) dot. More precisely, in the chase-present (but not the chase-absent) trials, the wolf displayed chasing behavior defined by the degree to which the dot reliably moved towards the sheep (chasing subtlety). Next to this baseline condition, in which we only manipulated chasing subtlety, we included (1) a social condition, in which eyes were added to all shapes and those of the wolf were always oriented towards the sheep, and (2) a non-social condition, in which wolf and sheep synchronously changed color (for example, see Fig. 1). While this social condition remained similar to previous orientation manipulations (e.g., whether and how the shapes, usually arrows, faced each other in the chasing display) (for review, see^35^), the non-social condition was an entirely new stimulus manipulation. Both conditions provided additional, meaningful information on the presence/absence of a chase, which we could compare to the baseline condition where the interdependency of the spatio-temporal trajectories of two dots was the only stimulus cue available. Whether the spontaneous perception of the interdependency as a social act of chasing also helps is not really known. By adding the eyes we could explicitly test the role of social interpretations, which we could compare to the baseline condition with only spontaneous, implicit social interpretation and to the non-social condition where the chase was cued differently. By these comparisons, we wanted to test whether animacy perception occurs automatically and reflects visual processing specialized for the extraction of animacy from visual motion rather than more conscious, higher-level interpretations^35^. We thereby predicted that TD participants would perform better when adding a socially-salient (e.g., eyes), compared to a less socially-salient (e.g., color change), cue to the display, due to the stronger social saliency of the interacting objects in this social condition. Finally, we were also interested in determining (1) whether adolescents with ASD, who display problems in social adaptation in everyday life, would perform worse than TD adolescents in a task using an implicit measure of agency detection and (2) whether adolescents with ASD would make use of social or non-social cues to the same extent as TD adolescents. Based on previous research^19,21^, we predicted that adolescents with ASD, compared to TD adolescents, would show less spontaneous responsivity to the socially-relevant information during chase detection. As a result, although we expected adolescents with ASD already to perform worse during implicit agency detection, this difference was assumed to be larger when adding the social, but not the non-social, cue to the chasing display.

## Results

**Figure 2:**
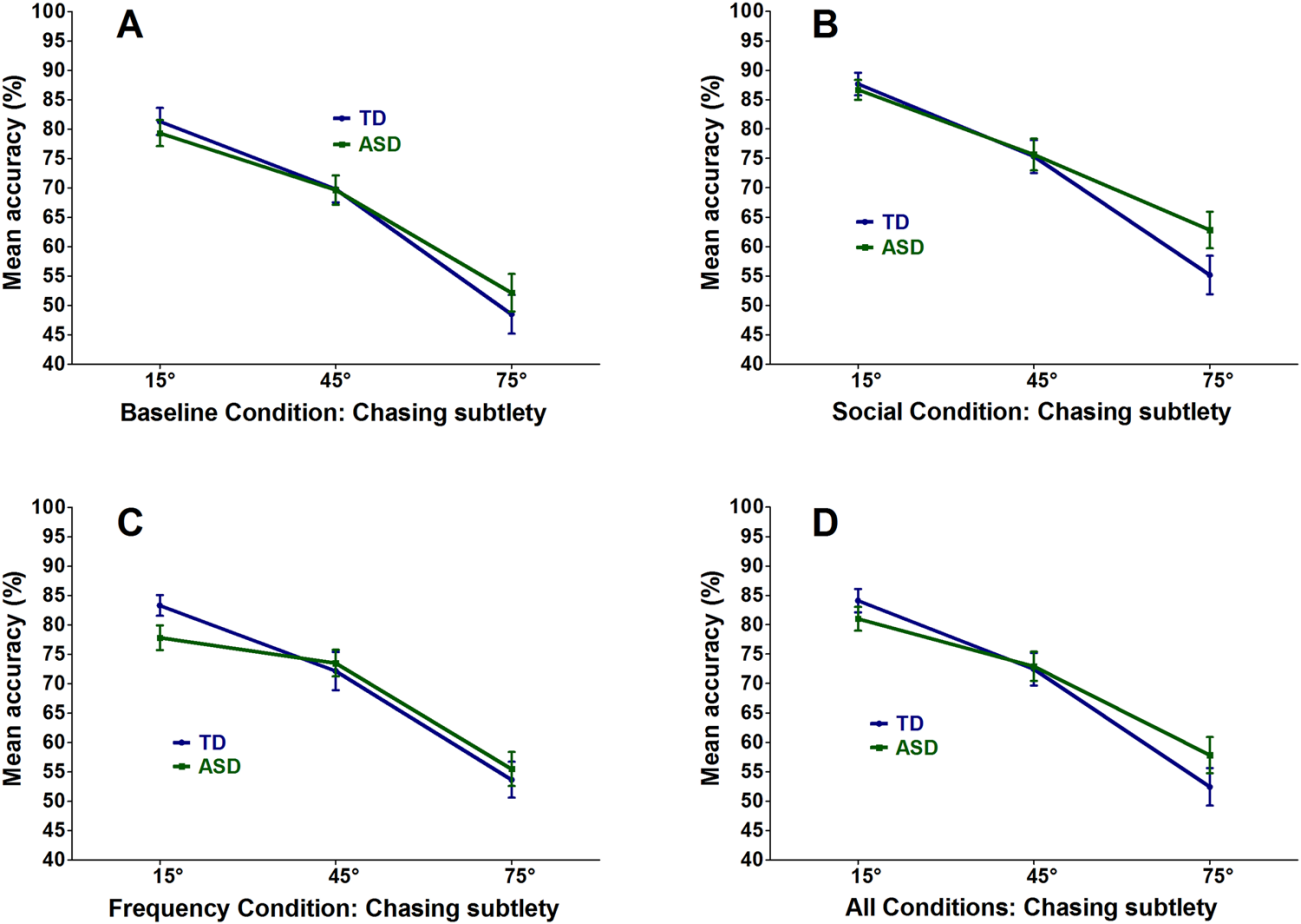
Overview of the mean accuracy performance. The data are represented as the mean performance across participants, with error bars depicting the standard error of the mean (*SEM*). The data of (**A**) the baseline condition, (**B**) the social condition, (**C**) the non-social condition and (**D**) all conditions combined, each time presented for all three subtlety values (15°, 45°, 75°). TD adolescents are depicted in blue and adolescents with ASD in green.

The GLMM analysis (for parameter estimates and 95% confidence intervals, see Table 1) indicated a significant main effect of Subtlety (*Z* = −11.54; *p* < 0.001). This showed that all participants were better at the task when the maximal angular deviation of the heading of the wolf was smaller or closer to perfect heat-seeking behavior (Fig. 2). This was a replication of previous findings underlining the influence of chasing subtlety on chase detection performance^13^. Furthermore, we also found a significant main effect, compared to the baseline condition, of adding social information to the display (*Z* = 6.11; *p* < 0.001). We did not find a main effect of adding non-social information to the display (*Z* = 1.20; *p* = 0.23). Furthermore, we observed a significant main effect of Group (*Z* = −2.06; *p* = 0.04), combined with a significant Group x Subtlety (*Z* = −1.97; *p* = 0.05) interaction effect. This interaction (Fig. 2D) indicated that adolescents with ASD performed worse than TD adolescents when the chase display closely resembled perfect heat-seeking behavior (e.g., 15°), while the opposite pattern emerged (ASD > TD) when the maximal angular deviation of the heading of the chasing dot became larger (e.g., 75°). More concretely, when also taking the main effect of Group into account, the model specifically indicated that the adolescents with ASD performed worse at a chasing subtlety of 15°, similar at 45° and better at 75° than TD adolescents. This means that the performance of adolescents with ASD depended less on chasing subtlety than that of their TD counterparts. Interestingly, we did not observe a significant Group x Condition interaction (not withheld during GLMM model selection, see Supplementary materials). This indicated that adolescents with and without ASD did not differ in their use of the added social or non-social cues.

**Table 1:** Overview of the parameter estimates for the chase detection task for the random intercepts logistic regression analysis on the trial-by-trial accuracy data.

RT			
Parameter	Estimate (Standard Error)	p-value	95% confidence interval
Intercept	−1.13 (0.98)	0.25	[−3.05; 0.79]
Group	−0.39 (0.19)	0.04	[−0.76; −0.02]
Age	0.17 (0.07)	0.01	[0.03; 0.31]
Trial type (chase-absent/present)	0.23 (0.14)	0.09	[−0.04; 0.50]
Subtlety	−0.05 (4.38 * 10^−3^)	<0.001	[−0.06; −0.04]
Social Condition	0.66 (0.11)	<0.001	[0.44; 0.88]
Non-social Condition	0.15 (0.13)	0.23	[−0.11; 0.41]
Group x Subtlety	0.01 (5.10 * 10^−3^)	0.05	[4.00 * 10^−6^; 02]

**Figure 3:**
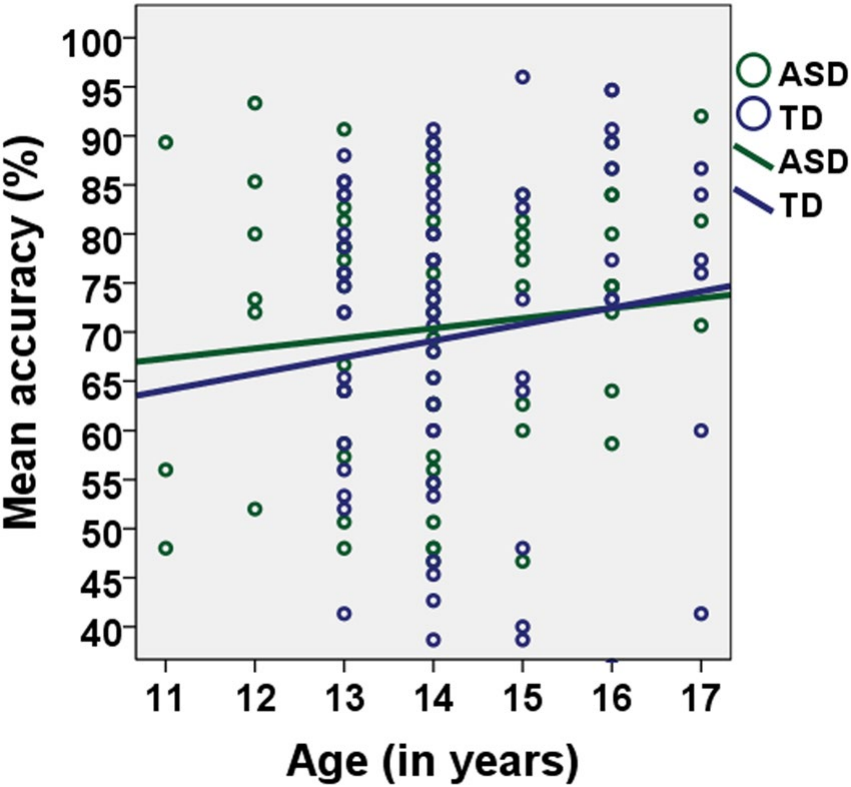
Visualization of the data, with the linear (per group) regression line indicating the strength of the correlation between the mean accuracy (%) on the ordinate axis and age (in years) on the abscissa. TD adolescents are depicted in blue and adolescents with ASD in green.

Finally, we found a significant influence of Age (*Z* = 2.61; *p* = 0.01) on participant performance (Fig. 3). This indicated that the chase detection of older adolescents, with and without ASD, was better than that of their younger counterparts. None of the other participant or task characteristics elicited an improvement in predicting chase detection performance.

## Discussion

The first goal of our study was to replicate the findings of Gao and colleagues^13^ on animacy perception in TD participants. In line with this study, our results indicated that chasing subtlety strongly affected chase detection performance. This could be interpreted as evidence for animacy perception to occur automatically and to reflect visual processing (rather than more conscious, higher-level interpretations) specialized for the extraction of animacy from visual motion (for review, see^35^). More generally, the authors emphasized that the purpose of vision is not only to recover the physical structure of the local environment, but also to recover its causal (and social) structure^36^. This was in accordance with the idea that not only physical properties of objects are accessed directly (and automatically) by perceptual processes, but also their intentional and/or social properties^37^. Furthermore, as an extension of the research of Gao and colleagues^13^, we found that chasing performance only improved when a perceptually-salient social cue, but not when a less perceptually-salient, or less naturalistic, non-social cue, was added to the task. This indicated a strict dependency of animacy perception on subtle visual display details, which were hypothesized to be most effective when automatically reinforcing the social saliency of the interacting objects (e.g., eyes).

All participants, with and without ASD, were able to successfully perform the chase detection task. Furthermore, no significant group-level differences were found with regard to the overall performance pattern when comparing the three separate conditions with each other (Baseline, Social and Non-social Condition). This was in contradiction with our initial hypothesis, expecting adolescents with ASD, compared to TD participants, (1) to perform worse in a task using an implicit measure of agency detection and (2) to use the added social (e.g., eyes) or non-social (e.g., color change) information differently. Interestingly, we did observe that the chase detection performance of adolescents with ASD was less dependent on chasing subtlety than that of their TD counterparts.

### Chase detection as animacy perception

The absence of group-level differences in the use of additional non-motion cues indicated that adolescents with and without ASD were similarly responsive to the increased perception of animacy when adding ‘relevant’ social information. This might be explained by recent evidence, suggesting that the perceptual system of individuals with ASD returns functionally intact signals for interpreting goal-directed actions adequately, especially when these individuals are prompted and motivated to do so under controlled conditions^21,38^. Furthermore, the added social cue might have been interpreted as perceptually salient, and reliable, low-level stimulus information by individuals with ASD^39^. Using a more detailed attention-focused processing of the local (or dot) chase elements, coinciding or following an integration of the different dot trajectories (e.g., wolf and sheep), they might have been able to detect the matching changes in the environment (‘eye’ direction and dot movement) as meaningful low-level stimulus information. This could have allowed them to largely overcome their, compared to TD individuals, reduced spontaneous responsivity to socially-relevant information in this task^21,40^. The absence of a chase detection advantage in the non-social condition for adolescents with ASD could then be explained by the less perceptually-salient nature of the color change manipulation. Furthermore, we did observe that the performance of adolescents with ASD depended less on chasing subtlety than that of their TD counterparts. The (slightly) better performance of TD adolescents in trials closely resembling perfect heat-seeking behavior, could indicate that the TD adolescents still had a stronger spontaneous interpretation of animacy than the adolescents with ASD. With higher chasing subtlety, the implicit social interpretation of the chase display lost its saliency and a more attention-focused processing of the chase elements gradually became a better search strategy. This could explain why individuals with ASD were better than the TD adolescents in the more difficult chase detection trials (75°). To conclude this section, if indeed the chasing task is a good implicit measure of spontaneous social inference as argued by Gao and colleagues^13^, intact performance in ASD seems inconsistent with cognitive accounts of ASD centering on impaired (implicit) social cognition^2^.

### Chase detection as visual search

An alternative interpretation of these findings relates to recent evidence suggesting that chase detection is not effortless^41,42^. More precisely, these studies argued that chase detection requires an object-based attentional selection of chase-relevant stimulus information with clear performance costs when, e.g., increasing the set size of the chasing display or manipulating the usefulness of spatial proximity between wolf and sheep to evaluate chase presence/absence. Interestingly, in these studies, observers were found not to select pairs of objects during chase detection, but to evaluate the motion pattern of a single object to decide whether or not it was involved in a chase. This made the authors conclude that chase detection requires an effortful, item-by-item, visual search through the subsets of all possible items in the display. As a consequence, the chase detection paradigm would be mostly comparable with a visual conjunction search task, in which focused visual attention is needed to serially scan and integrate the display elements into a consistent whole (object)^43,44^. To correctly detect the presence/absence of a chase, participants have to combine motion and stimulus (eyes, color change) information effectively. Within this interpretation of our findings, it might be that our ‘social’ and ‘non-social’ display manipulation mainly differed in terms of perceptual saliency and not in terms of social meaningfulness. The absence of a condition-specific group-level difference in performance could therefore indicate that the three conditions did not strongly differ in search difficulty, although the so-called ‘social’ condition was easier due to the extra perceptually-salient cue information. Furthermore, the weaker dependency on chasing subtlety in ASD might relate to their enhanced performance in complex visual search tasks^45,46^. As in visual search, the enhanced performance in ASD became especially apparent in the more difficult chase detection trials. This further argued for an item-by-item search through the different elements of the chase display, instead of an automatic processing of the social saliency of the interacting objects. This explanation of intact (or even improved) chase detection in terms of perceptual and attentional mechanisms may be consistent with accounts of ASD that assume improved sensitivity for local deviations^39^, although the extent to which the task can be performed on purely local grounds still merits further investigation. One such local, non-social cue might be the inter-object spacing between chaser and chasee^41,47^. During a chase, the distance between the pair of chasing objects decreases relative to the non-chasing objects (even at larger subtleties). Individuals with ASD might be more sensitive to this type of spatial proximity cue than their TD counterparts (making them perform better than their TD counterparts during chase detection with higher chasing subtleties).

Finally, another explanation for why chasing subtlety could work differently between the two groups is based on the rationality principle^48^. It states that, to be perceived as animate and goal-directed, an agent needs to achieve a goal with maximum efficiency. This can explain why an observer detects chasing only when the wolf pursuits the sheep with heat-seeking motion, persistently over time. Following this rationality principle, one should readily detect small subtlety values but ignore larger subtlety values. Hence, better performance of the ASD group in the high subtlety condition may suggest that their perceived chasing is less constrained by the rationality principle.

### Future research

The intact performance of adolescents with ASD in this task seemed inconsistent with cognitive accounts of ASD centering on impaired (implicit) social cognition. We were therefore not able to make strong claims about the chasing task as being a good implicit measure of spontaneous social inference as argued by Gao and colleagues^13^. However, the social and the non-social conditions in our study might differ in other aspects than the intended ones. For instance, the ‘eyes’ of the wolf provide a constant pointer towards the sheep whereas the simultaneous sinusoidal color changes in the non-social condition do not provide such spatial information. Future research using the current chase detection paradigm should therefore also test different, less spatially-defined, manipulations of social saliency (e.g., restricting the differences between the social and non-social chase detection to the cover story, providing a socially-salient versus socially-neutral chasing background,…). Furthermore, previous research indicated that measuring eye movements during chase detection (or other animated displays) could provide useful (and implicit) tools to systematically assess the degree of mental state attribution in different social versus non-social experimental conditions while taking low-level kinematic confounds (e.g., the low-level physical stimulus properties of the different experimental conditions) into account^49,50^. Future research may also benefit from using irrelevant and informative chase detection cues, each with varying reliabilities, in order to better approach naturalistic social settings that usually cause problems in ASD^51^. Deciphering the stimuli and/or display properties influencing the presence and strength of implicit social inferences within such a laboratory-controlled experimental setting could then provide meaningful insights to identify the corresponding characteristics of complex everyday life situations. This translation from abstract, laboratory-controlled, experiments to rich social scenes, involving multiple concurrent salient cues, as in eye contact and communicative interaction, could be especially important to better understand the social problems encountered in ASD^33,52^.

## Conclusion

In this study we intended to explore the strength of implicit social inferences in adolescents with and without ASD using a chasing paradigm, similar to Gao and colleagues^13^, in which participants judged the absence or presence of a chase within a display of seemingly random moving dots. Our results indicated that, in line with the original findings, chasing subtlety strongly affected chase detection performance. Furthermore, we also found that chase detection only improved when we added social, but not non-social, information to the display. Interestingly, adolescents with and without ASD did not differ in their use of the added social (or non-social) cue. However, the chase detection performance of the adolescents with ASD was intact and depended less on chasing subtlety than that of their TD counterparts. This may relate to differences in visual search performance, which plays a stronger role in the more challenging (high chasing subtlety) search displays.

## Methods and materials

### Participants

A group of 24 adolescents (20 males) with ASD (mean age = 14.08; *SD* = 1.47) and a TD control group (mean age = 14.33; *SD* = 1.27), which were individually matched on age, gender and IQ, participated in this study (see Table 2 for participant characteristics). IQ was estimated using an abbreviated four-subtest (Vocabulary, Similarities, Picture Completion and Block Design) version of the WISC-III^53,54^. All participants also completed the Dutch Social Responsiveness Scale (SRS) questionnaire^55^ to get an overall estimation of individual and/or group-level differences in ASD traits.

**Table 2:** Overview of the average group-level performance (SD between brackets), for participants with ASD (*n* = 24) and TD participants (*n* = 24). On each of the descriptive tests a one-way ANOVA, with Bonferroni correction for multiple comparisons when required, with Group as between-participants factor was conducted.

Variable	TD adolescents	ASD adolescents	TD vs ASD	Effect size
**Age**	14.08 (1.47)	14.33 (1.27)	*F*_*1,46*_ = 0.40; *p* = 0.53	*η* ^2^ = 0.01
**Full-Scale IQ**	103.44 (10.23)	105.63 (9.05)	*F*_*1,46*_ = 0.62; *p* = 0.44	*η* ^2^ = 0.01
**Verbal IQ**	102.38 (15.76)	105.96 (7.53)	*F*_*1,46*_ = 1.01; *p* = 0.32	*η* ^2^ = 0.02
**Performal IQ**	107.29 (15.37)	105.29 (13.28)	*F*_*1,46*_ = 0.23; *p* = 0.63	*η* ^2^ < 0.01
**SRS (Overall)**	81.04 (12.97)	49.67 (8.07)	*F*_*1,46*_ = 101.22; *p* < 0.001	*η* ^2^ = 0.69

The adolescents with ASD all had a formal clinical diagnosis of ASD and were diagnosed according to DSM-IV-TR criteria^56^ in a multidisciplinary team. Recruitment was set up via the Autism Expertise Centre of the University Hospital in Leuven. Furthermore, a trained clinical psychologist administered the Dutch version of the Autism Diagnostic Observation Schedule 2 (ADOS-2) module 3 from all participants with a clinical diagnosis. ASD diagnoses were re-confirmed in 22 of the 24 adolescents, with the new ADOS Algorithm for DSM-IV/ICD-10 (ADOS-2). Since the analyses did not differ depending on whether we in- or excluded the participants scoring below the ADOS-2 cut-off score, we followed the clinical diagnosis of the participants and reported the results of the full ASD group. All participants had normal or corrected-to-normal vision.

The study was approved by the Medical Ethics Commission of KU Leuven and all participants, and their parents, provided written informed consent before onset of the experiment. Furthermore, our study was performed in accordance with the Declaration of Helsinki and written informed consent was obtained according to institutional guidelines of the local research ethics committee. All the participants were debriefed and thanked following their participation.

### Stimuli and procedure

Participants were seated at 57 cm from the calibrated (gamma corrected) computer monitor (resolution: 1920 × 1200; refresh rate: 60 Hz; type: Monitor DELL U2410) in a dimly lit room. The task took about 30 minutes, all instructions were provided on the computer screen, and every trial started with a 2-second countdown before the actual chasing display was presented. This display contained four identical moving circular shapes (apparent size: 1° of visual angle), shown for 3000 ms. At the start of each trial, three of the four shapes (dots) started moving at a constant speed of 14.5°/s and moved haphazardly by randomly changing direction within a 120° window (approximately every 170 ms). The fourth, and final, dot was termed the ‘wolf’ and moved differently. More precisely, the heading of the wolf towards the sheep was manipulated by altering its maximal angular deviation compared to perfect “heat-seeking” behavior (chasing subtlety). We implemented three different chasing subtleties: 15°, 45°, and 75°. When the chasing subtlety was 15° (or 45° or 75°), for instance, the wolf was always heading in the general direction of the sheep, but was not perfectly heat-seeking. The dot could move in any direction within a 30° (or 90° or 150°) window, with the window always centered on the (moving) sheep. In chase-absent trials this wolf chased an invisible fifth dot, the sheep, while in chase-present trials the wolf chased one of the three visible dots (in the role of sheep) on the screen. These trajectories were generated so that the wolf-sheep distance always exceeded 5°. In order to retain this minimum distance, the wolf maintained its direction of motion in those instances in which a direction change would result in a violation of the wolf-sheep minimum distance (similar to^47^). The same behavior was also shown by the other shapes in the display when they had to avoid a ‘collision’ with another shape (objects were not allowed to hit, or to cross, each other). As a result, the movement pattern of the wolf was very similar to that of the other shapes and only differed slightly in the amount of direction changes (due to the larger space restriction with regard to the sheep). Furthermore, the chasing behavior of the wolf was delayed for 170 ms at the start of each trial (allowing the wolf to follow a more natural – less ‘bumpy’ or curved – chasing pattern). As a result, the only factor that differed between the chase-absent and chase-present trials was whether the sheep being chased was visible in the display or not. At the end of each trial (after the 3-second animation), the shapes disappeared from the screen and the participant was required, without time constraints, to indicate whether or not the trial contained a chase. If participants correctly identified a chase-present display, they also indicated where the sheep was last seen in the display by clicking on one of the four quadrants of the computer screen. This was done to motivate the adolescent participants not to guess or respond randomly during the chase detection task and was not intended to be included in the formal analysis afterwards given the very imprecise nature (e.g., screen quadrants) of the dot localization.

All participants had to complete three blocks (conditions) presented in a counterbalanced order: In the *baseline condition* we only manipulated the chasing subtlety. In the *social condition*, all three chasing subtleties were also present, but eyes were added to the shapes and the eyes of the wolf were always oriented towards the sheep in the case of chase-present trials. Finally, in the *non-social condition*, in addition to the chasing subtlety, we sinusoidally varied the color of the shapes (with a phase offset of 0.15 radians and an amplitude of 1). For the chase-present trials the frequency of the color change of the wolf and the sheep was the same (3 Hz), while the color of the two distractor dots in the display varied at a different frequency (0.5 Hz). All participants completed 75 trials per condition (25 per chasing subtlety, of which 20 chase-present and 5 chase-absent trials). Each condition started with a total of 6 practice trials, both a chasing-present and -absent trial for each chasing subtlety, with visual feedback.

### Analyses

We analyzed the accuracy (correct/incorrect) scores using General Linear Mixed Modeling (GLMM)^57^ and, based on the dichotomous nature of the dependent variable, we chose to use a logistic regression modelling approach (for an overview, see Supplementary materials). After model selection, the individual predictive value of each selected parameter was tested using Wald Z-tests^58^. Participant was always regarded as random intercept and both participant (e.g., FSIQ, SRS and age) and task (e.g., test order and trial type (chase-absent versus chase-present trials)) characteristics were tested as possible covariates. The results of this analysis are reported in the results section of the manuscript and more details on the model selection process, in terms of fixed effects and goodness-of-fit measures, are provided in the Supplementary materials. Furthermore, we also evaluated possible group-level differences by calculating both the mean accuracy and sensitivity (*d’*) values for each of the participants as a dependent variable in a mixed ANOVA with Group (ASD versus TD) as between-subjects factor, Condition (baseline, social, non-social) and Subtlety (15°, 45°, 75°) as within-subjects factors and Participants as a random factor. The mixed ANOVA analysis of both the mean accuracy and the *d’* values provided very similar results as the GLMM approach. Furthermore, given the low amount of False Alarms (FA) trials per participant in each subtlety and condition, the calculation of the FA ratio was conducted using different averaging criteria (for overview, see Supplementary materials), which were found to affect the calculation of the *d’* prime values. We therefore decided only to report the GLMM outcomes and to include all other results (ANOVA on mean accuracy, *d’*) in the Supplementary materials. All analysis were conducted using the statistical software programs R (version 3.1.1) and IBM SPSS (version 22).

### Data availability statement

The datasets generated during and/or analyzed during the current study are available from the corresponding author on reasonable request.

## Electronic supplementary material

Supplementary information
