# Supplementary material for: Intact animacy perception during chase detection in ASD
Source: Sci Rep. 2017 Sep 19;7:11851. doi: 10.1038/s41598-017-12204-x (PMC5605503; doi:10.1038/s41598-017-12204-x)
Supplement: Supplementary file 1 — Supplementary information [file 41598_2017_12204_MOESM1_ESM.doc]

*Supplementary materials for*

Intact animacy perception during chase detection in ASD

Steven Vanmarcke^1,2*^, Sander van de Cruys^1,2^, Pieter Moors^1^, Johan Wagemans^1,2^

(1) Brain and Cognition, KU Leuven, Leuven, 3000, Belgium

(2) Leuven Autism Research (LAuRes), KU Leuven, Leuven, 3000, Belgium

**A. Mixed ANOVA (mean accuracy)**

**Analysis**

To evaluate possible group-level differences, we calculated the mean accuracy for each of the participants as a dependent variable in a mixed ANOVA with Group (adolescents with versus without ASD) as between-subjects factor and Condition (baseline, social, frequency) and Subtlety (15°, 45°, 75°) as within-subjects factors. Participants were regarded as a random factor.

**Results**

The mixed ANOVA analysis of the data (Table 1Sup) indicated that there was a significant main effect of both (1) Subtlety (*F_2,92_* = 243.58; *p* < .001; *η^2^* = .84) and (2) Condition (*F_2,92_* = 13.60; *p* < .001; *η^2^* = .23). The former finding, (1), showed that all participants were better at the task when the maximal angular deviation of the heading of the chasing dot was smaller or closer to perfect heat seeking behavior. This was a direct replication of previous findings underlining the influence of chase subtlety on chase detection performance (Gao et al., 2009). The latter observation, (2), further indicated that the different conditions had a differential influence on performance. More precisely, when conducting all pairwise contrasts (Bonferroni corrected for multiple comparisons), we found that the social condition significantly differed from both the baseline (*F_1,46_* = 24.53; *p* < .001; *η^2^* = .35) and the frequency (*F_1,46_* = 10.43; *p* < .01; *η^2^* = .19) condition. However, no significant differences were found between the baseline and frequency condition (*F_1,46_* = 3.69; *p* = .06; *η^2^* = .07). This implied that all adolescents performed better in the Social condition, compared to the baseline, but not in the Frequency condition.

Importantly, the analysis did not reveal a significant main effect of Group (ASD versus non-ASD) on participant performance. This indicated that the performance of adolescents with and without ASD, irrespective of chasing condition, did not differ. We only observed a significant Group x Subtlety interaction (*F_2,92_* = 3.56; *p* = .03; *η^2^* = .07) effect. This finding implied that adolescents with ASD performed differently than TD participants at different levels of chase subtlety, irrespective of chasing condition.

Table 1Sup. Outcomes of the mixed ANOVA with Group (adolescents with versus without ASD) as between-subjects factor and Condition (baseline, social, frequency) and Subtlety (15°, 45°, 75°) as within-subjects factors.

|  | | | |
| --- | --- | --- | --- |
| **Parameter** | **F-statistic** | **p-value** | **Effect size (*η^2^_p_*)** |
| Condition | F_2,92_ = 13.60 | < .001 | *η^2^* = .23 |
| Subtlety | F_2.92_ = 243.58 | < .001 | *η^2^* = .84 |
| Group | F_1,46_ = .09 | .76 | *η^2^* < .01 |
| Group x Condition | F_2,92_ = .67 | .52 | *η^2^* = .01 |
| Group x Subtlety | F_2,92_ = 3.56 | .03 | *η^2^* = .07 |
| Condition x Subtlety | F_4,184_ = 1.40 | .24 | *η^2^* = .03 |
| Group x Condition x Subtlety | F_4,184_ = .81 | .52 | *η^2^* = .02 |

**B. Mixed ANOVA (sensitivity)**

**Analysis**

To evaluate possible group-level differences, we calculated the sensitivity (*d’*) for each of the participants as a dependent variable in a mixed ANOVA with Group (adolescents with versus without ASD) as between-subjects factor and Condition (baseline, social, frequency) and Subtlety (15°, 45°, 75°) as within-subjects factors. Participants were regarded as a random factor. We choose *d’* as an extra, alternative dependent variable for mean accuracy because the monotonic function provides an indication of the performance for each observer, by combining the Hit (H) rate (proportion correctly judged go trials) with the False Alarm (FA) rate (proportion incorrectly judged no go trials) into a single standardized score: *d’* = Z[H] – Z[FA]. Within this framework, Z corresponds to the inverse of the normal distribution function. Nonetheless, given the low amount of FA trials per participant in each subtlety and condition, we decided to calculate *d’* using four different calculation criteria of the FA ratio: (1) averaging over all FA trials per participant, (2) averaging over all FA per condition per participant, (3) averaging over all FA per subtlety per participant and (4) using only the FA trials provided per condition per subtlety per participant. We simultaneously checked whether this (choice of) FA calculation directly influenced our outcomes by also adding FA calculation Method ( (1), (2), (3), (4) ) as a between-subjects factor in a separate analysis of the data.

**Results**

The mixed ANOVA analysis of the data (provided per FA calculation method in Table 2Sup) indicated that the choice of FA calculation had a significant impact on the calculation of the *d’* prime values (*F_3,184_* = 7.06; *p* < .001; *η^2^* = .10). This argued against using the standardized *d’* prime scores as the main dependent variable in the current experiments. We believe that this observation follows from calculating *d’* based on a very limited amount of FA trials per participant, in each separate subtlety and for each condition, which increases the amount of variability in the individual *d’* scores. More precisely, in the current analysis the lowest standard deviation was observed when calculating d’ by averaging over all FA trials (45) per participant (*SD* = .84). When averaging over all FA trials (15) per condition per participant (*SD* = 1.06) or averaging over all FA trials (15) per subtlety per participant (*SD* = 1.01), the standard deviation in d’ prime scores increased substantially. Finally, the largest variability in scores was obtained when using only the FA trials (5) provided per condition per subtlety per participant (*SD* = 1.45).

Nonetheless, and most importantly, our findings remained very similar to the outcomes of the mixed ANOVA on the mean accuracy values. In all analysis, we found both significant main effects for (1) Subtlety and (2) Condition. The former finding, (1), showed that all participants were better at the task when the maximal angular deviation of the heading of the chasing dot was smaller or closer to perfect heat seeking behavior. This was a direct replication of previous findings underlining the influence of chase subtlety on chase detection performance (Gao et al., 2009). The latter observation, (2), further indicated that the different conditions had a differential influence on performance. More precisely, when conducting all pairwise contrasts (Bonferroni corrected for multiple comparisons), we found that the social condition always significantly differed from both the baseline and the frequency condition. However, no significant differences were found between the baseline and frequency condition. This implied that all adolescents performed better in the Social condition, compared to the baseline, but not in the Frequency condition.

Interestingly, none of the analysis revealed a significant main effect of Group on participant performance. This indicated that the performance of adolescents with and without ASD, irrespective of chasing condition, did not differ. We only observed a significant Group x Subtlety interaction effect in the most data-driven FA calculation method (per participant, per subtlety and per condition). This finding was in line with the mixed ANOVA on the mean accuracy data and implied that adolescents with ASD might perform differently than TD participants at different levels of chase subtlety, irrespective of chasing condition.

Table 2Sup. Outcomes of the mixed ANOVA with Group (adolescents with versus without ASD) as between-subjects factor and Condition (baseline, social, frequency) and Subtlety (15°, 45°, 75°) as within-subjects factors. Given the low amount of FA trials per participant in each subtlety and condition, we decided to calculate *d’* using four different calculation criteria of the FA ratio: (1) averaging over all FA trials per participant, (2) averaging over all FA per condition per participant, (3) averaging over all FA per subtlety per participant and (4) using only the FA trials provided per condition per subtlety per participant.

| **FA calculation** | | | | |
| --- | --- | --- | --- | --- |
| **Parameter** | **Per participant** | **Per participant, per condition** | **Per participant, per subtlety** | **Per participant, per subtlety and per condition** |
| Condition | *F_2,92_* = 15.30; *p* < .001;  *η^2^* = .25 | *F_2,92_* = 9.14; *p* < .001;  *η^2^* = .17 | *F_2,92_* = 15.30; *p* < .001; *η^2^* = .25 | *F_2,92_* = 15.38; *p* < .001;  *η^2^* = .14 |
| Subtlety | *F_2,92_* = 260.69; *p* < .001; *η^2^* = .85 | *F_2,92_* = 260.70; *p* < .001;  *η^2^* = .85 | *F_2,92_* = 43.06; *p* < .001; *η^2^* = .48 | *F_2,92_* = 47.72; *p* < .001;  *η^2^* = .51 |
| Group | *F_1,46_* = .69; *p* = .41;  *η^2^* = .02 | *F_1,46_* = 1.02; *p* = .32;  *η^2^* = .02 | *F_1,46_* = .62; *p* = .44;  *η^2^* = .01 | *F_1,46_* = 1.10; *p* = .30;  *η^2^* = .02 |
| Group x Condition | *F_2,92_* = .07; *p* = .93;  *η^2^* < .01 | *F_2,92_* = 2.01; *p* = .14;  *η^2^* = .04 | *F_2,92_* = .07; *p* = .93;  *η^2^* < .01 | *F_2,92_* = 1.73; *p* = .18;  *η^2^* = .04 |
| Group x Subtlety | *F_2,92_* = .87; *p* = .42;  *η^2^* = .02 | *F_2,92_* = .86; *p* = .42;  *η^2^* = .02 | *F_2,92_* = 1.47; *p* = .24;  *η^2^* = .03 | *F_2,92_* = 3.05; *p* = .05;  *η^2^* = .06 |
| Condition x Subtlety | *F_4,184_* = 2.28; *p* = .06;  *η^2^* = .05 | *F_4,184_* = 2.28; *p* = .06;  *η^2^* = .05 | *F_4,184_* = 2.28; *p* = .06;  *η^2^* = .05 | *F_4,184_* = .84; *p* = .50;  *η^2^* = .02 |
| Group x Condition x Subtlety | *F_4,184_* = .60; *p* = .66;  *η^2^* = .01 | F_4,184_ = .60; *p* = .66;  *η^2^* = .01 | *F_4,184_* = .60; *p* = .66;  *η^2^* = .01 | *F_4,184_* = 1.01; *p* = .40;  *η^2^* = .02 |

**C. Results of the GLMM model selection process**

We analyzed the accuracy (correct/incorrect) scores using General Linear Mixed Modeling (GLMM) and, based on the dichotomous nature of the dependent variable, we choose to use a logistic regression modelling approach. Based on a maximum likelihood estimation, we calculated the deviance values for the different random intercepts logistic regression models and selected the final model by evaluating the drop in deviance, together with the Akaike and Bayesian Information Criterion values. More precisely, we regarded the former as our main criterion for model selection, while the latter two information criterions provided extra information. In the added table (Table 2Sup), we provided our results in terms of fixed effects and goodness-of-fit measures, with the Drop in Deviance value always comparing the previous and the consecutive model (e.g., Model A versus Model B,…).

Table 2Sup. Outcomes of the GLMM model selection process in terms of fixed effects and goodness-of-fit measures.

| **Fixed effects** | | | **Model A** | | **Model B** | | **Model D** | **Model E** | **Model G** | **Model H** |
| --- | --- | --- | --- | --- | --- | --- | --- | --- | --- | --- |
|  | Intercept | | -.44***  (.12) | | 1.44***  (7.36 * 10^-4^) | | 1.02***  (.12) | 1.37***  (.21) | -.83  (2.47) | -1.13  (.98) |
|  | Subtlety | | --- | | -.04***  (7.00 * 10^-4^) | | -.04***  (4.67 * 10^-3^) | -.05***  (5.69 * 10^-3^) | -.07*  (.03) | -.05***  (4.38 * 10^-3^) |
|  | Social Condition | | --- | | --- | | .62***  (.10) | .70*  (.27) | 1.22  (1.18) | .66***  (.11) |
|  | Frequency Condition | | --- | | --- | | .12  (.12) | .20  (.27) | .98  (1.22) | .15  (.12) |
|  | Group | | --- | | --- | | -.42**  (.16) | -.19  (.29) | .93  (3.05) | -.39*  (.19) |
|  | Age | | --- | | --- | | --- | --- | .22*  (.11) | .17**  (.06) |
|  | FSIQ | | --- | | --- | | --- | --- | -.02  (.02) | --- |
|  | SRS | | --- | | --- | | --- | --- | .02  (.02) | --- |
|  | Trial type (chase-absent/present) | | --- | | --- | | --- | --- | .12  (.19) | .23  (.14) |
|  | Test order | | --- | | --- | | --- | --- | .07  (.08) | --- |
|  | Group x Subtlety | | --- | | --- | | .01*  (6.64 * 10^-3^) | 7.13 * 10^-3^  (7.69 * 10^-3^) | 7.61 * 10^-3^  (8.15 * 10^-3^) | .01*  (5.10 * 10^-3^) |
|  | Group x Social Condition | | --- | | --- | | --- | -.24  (.38) | -.07  (.39) | --- |
|  | Group x Frequency Condition | | --- | | --- | | --- | -.47  (.38) | -.42  (.38) | --- |
|  | Group x Age | | --- | | --- | | --- | --- | -.09  (.13) | --- |
|  | Group x FSIQ | | --- | | --- | | --- | --- | .02  (.02) | --- |
|  | Group x SRS | | --- | | --- | | --- | --- | -.04  (.02) | --- |
|  | Group x Trial type | | --- | | --- | | --- | --- | -.65  (.34) | --- |
|  | Group x Test order | | --- | | --- | | --- | --- | .05  (.11) | --- |
|  | Subtlety x Social Condition | | --- | | --- | | --- | -3.86 * 10^-4^  (6.49 * 10^-3^) | 1.68 * 10^-3^  (6.60 * 10^-3^) | --- |
|  | Subtlety x Frequency Condition | | --- | | --- | | --- | 3.36 * 10^-3^  (6.65 * 10^-3^) | 3.58 * 10^-3^  (9.13 * 10^-3^) | --- |
|  | Subtlety x Age | | --- | | --- | | --- | --- | 1.02 * 10^-3^  (2.04 * 10^-3^) | --- |
|  | Age x Social Condition | | --- | | --- | | --- | --- | -.04  (.08) | --- |
|  | Age x Frequency Condition | | --- | | --- | | --- | --- | -.06  (.08) | --- |
|  | Group x Social Condition x Subtlety | | --- | | --- | | --- | 6.14 * 10^-3^  (8.92 * 10^-3^) | 1.77 * 10^-3^  (9.10 * 10^-3^) | --- |
|  | Group x Frequency Condition x Subtlety | | --- | | --- | | --- | 6.15 * 10^-3^  (9.02 * 10^-3^) | 3.58 * 10^-3^  (9.13 * 10^-3^) | --- |
|  |  | |  | |  | |  |  |  |  |
| **Goodness – of - fit** | |  | |  | |  | | | | |
|  | Deviance | | 12920.5 | | 12264.7 | | 12222.2 | 12211.5 | 12186.3 | 12202.7 |
|  | Drop in Deviance | | --- | | 44.2*** | | 42.5*** | 10.7 | 25.2* | -16.4 |
|  | Aikake Information Criterion | | 12924.5 | | 12274.7 | | 12246.2 | 12247.5 | 12248.3 | 12230.7 |
|  | Bayesian Information Criterion | | 12939.0 | | 12311.2 | | 12333.6 | 12378.7 | 12474.2 | 12332.7 |

Note. Overview of the used abbreviations: FSIQ = Full-Scale Intelligence Quotient, SRS = Social Responsiveness Scale.

For the fixed effects: * *p* < 0.05. ** *p* < 0.005. *** *p* < 0.0001
